# Hybrid EEG-fNIRS Asynchronous Brain-Computer Interface for Multiple Motor Tasks

**DOI:** 10.1371/journal.pone.0146610

**Published:** 2016-01-05

**Authors:** Alessio Paolo Buccino, Hasan Onur Keles, Ahmet Omurtag

**Affiliations:** 1 Department of Biomedical Engineering, University of Houston, Houston, Texas, United States of America; 2 Department of Electronics Informatics and Bioengineering, Politecnico di Milano, Milano, Italy; University of Minnesota, UNITED STATES

## Abstract

Non-invasive Brain-Computer Interfaces (BCI) have demonstrated great promise for neuroprosthetics and assistive devices. Here we aim to investigate methods to combine Electroencephalography (EEG) and functional Near-Infrared Spectroscopy (fNIRS) in an asynchronous Sensory Motor rhythm (SMR)-based BCI. We attempted to classify 4 different executed movements, namely, Right-Arm—Left-Arm—Right-Hand—Left-Hand tasks. Previous studies demonstrated the benefit of EEG-fNIRS combination. However, since normally fNIRS hemodynamic response shows a long delay, we investigated new features, involving slope indicators, in order to immediately detect changes in the signals. Moreover, Common Spatial Patterns (CSPs) have been applied to both EEG and fNIRS signals. 15 healthy subjects took part in the experiments and since 25 trials per class were available, CSPs have been regularized with information from the entire population of participants and optimized using genetic algorithms. The different features have been compared in terms of performance and the dynamic accuracy over trials shows that the introduced methods diminish the fNIRS delay in the detection of changes.

## Introduction

Brain-Computer Interfaces (BCI) try to extract information directly from the central nervous system in order to replace or supplement its output [1]. The primary technical goal of BCI research is to obtain the highest real-time information from brain activity in the most convenient and unobtrusive way, with the least setup time and calibration. The choice of measurement modality is therefore influenced by considerations such as equipment size and expense as well as the time and space resolution needed for specific applications. Current non-invasive methods include Electroencephalography (EEG), functional Near-Infrared Spectroscopy (fNIRS), functional Magnetic-Resonance-Imaging (fMRI), and Magnetoencephalograpy (MEG), each with its own advantages and limitations. EEG is a long established medical procedure that is sensitive to the organized synaptic activity of the brain. It is based on measuring voltage differences between electrodes placed on the scalp. EEG is currently the most actively used research tool in BCI, involving many different techniques (e.g. auto-regressive (AR) methods in [2–4], Wavelet transforms in [5–7], or Common Spatial Patterns in [8–10]). The main limitation of EEG lies in its spatial resolution, associated with the difficulty of localizing its sources [11, 12]. We utilized the hemodynamic signal measured by fNIRS as an additional source of information because its properties complement those of EEG and it is the only other non-invasive method that is practical and potentially mobile. In most fNIRS studies the use of two distinct wavelengths allows the extraction of the concentration changes of oxy- and deoxy-hemoglobin (HbO and HbR) in the outer layers of the cortex [13]. Following neural activation, local blood flow and volume typically increase on a time scale of seconds. Concentration changes measured by fNIRS result in a signal similar to the blood oxygen level dependent (BOLD) response obtained by fMRI [14, 15]. fNIRS technology has been used for BCI involving motor related paradigms from a number of groups [16–20]. The main limitation of fNIRS-based BCI appears to be the long lag that the hemodynamic response needs to reach its maximum, which makes it challenging to extract features usable in real-time application. Moreover, the possibility of using fNIRS to discriminate between multiple classes has not been investigated widely. At our knowledge, only one previous study investigated the possible benefit in EEG-fNIRS combination for a Sensory Motor Rhythm (SMR)-based BCI: Fazli et al. [21] showed that the performance of a hybrid BCI is enhanced when EEG features are combined with HbO and/or HbR derived features both for motor execution and motor imagery in a binary classification problem (Right-Hand—Left-Hand tasks). However, fNIRS-based classifiers showed an extensive delay (around 6–7.5 s) before reaching a peak in the accuracy. In this work we investigate the use of other methods to extract fNIRS features in order to limit the observed lag in the response. In particular, we applied two different approaches: one consisted of using Regularized Common Spatial Patterns (RCSP), and the other one involved the combination of average and slope indicators for the fNIRS signals, which have proved beneficial in previous studies [20]. Moreover, our study aims at investigating the recognition of 4 different classes (Right-Arm—Left-Arm—Right-Hand—Left-Hand) using an asynchronous paradigm [22], for which the user of the BCI communicates continuously with the machine without the need of a visual or auditory cue to pace the user in the communication. Such a BCI requires first of all the classification of Rest (no movement) or Task (any movement). The analyses were performed offline, but all the methods applied are designed to be easily applicable in a real-time setup.

The following section presents the setup and the design of the study, as well as the signal processing, feature extraction and classification approaches. In the Results section the experimental results are shown, with particular focus on the fNIRS temporal performance. Discussions and Conclusion section concludes the work by summarizing the findings and discussing their possible role in the field.

## Methods

### Experimental Design and Data Acquisition

**Fig 1:**
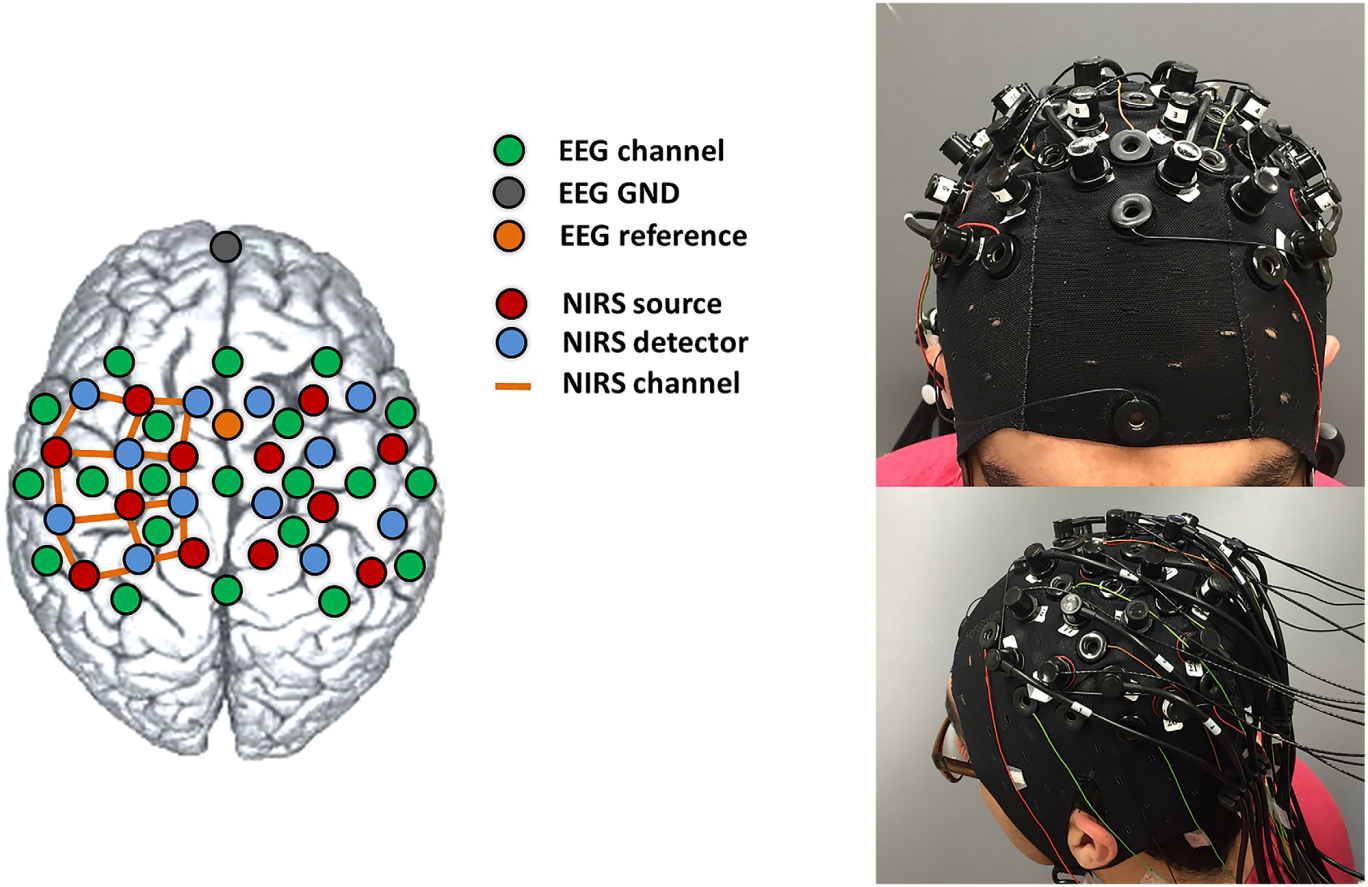
*Left*: EEG electrodes and fNIRS optodes configuration on the cap. *Right*: Real picture of a subject wearing the cap completely mounted (with EEG electrodes, fNIRS sources and detectors).

15 healthy right-handed male subjects, aged between 23 and 54 (average and standard deviation: 27.4±7.7), participated in the experiments, which lasted around 1 hour including the time required for the setup. Subjects completed and signed a written informed consent document before each experiment and were compensated for their participation. The research was approved by the Institutional Review Board (IRB) at University of Houston. The IRB approval included the consent procedure. During the experiment, subjects were seated in a comfortable chair and were asked to stay relaxed. The experiment consisted of 5 blocks of motor execution: in each block, subjects were to perform 20 trials divided in the 4 movements (Right-Arm—Left-Arm raising, and Right-Hand—Left-Hand gripping). The trials for each block were randomized, but the number of trials were evenly distributed, so that for each block 5 trials for each class were acquired. In total, 25 trials for each class were performed by each subject. The subjects were guided through the experiment following the visual instructions (textual) presented on a laptop screen placed around 1 m away from their eyes. Every trial started with 6 seconds of rest, and the subject was instructed for the following 6 seconds to move according to the screen direction (Right Arm, Left Arm, Right Hand, Left Hand) at a self-paced rhythm. Every trial lasted 12 seconds and the actual data acquisition was about 20 minutes excluding the instrumentation setup. Simultaneous EEG and fNIRS measurements were acquired during the experiment. The fNIRS system (NIRScout 8–16, NIRx Medizintechnik GmbH, Germany) was equipped with 12 sources and 12 detectors combined in 34 channels of acquisition. The channels were distributed evenly on the motor cortex, the sampling frequency was *f*_*nirs*_ = 10.42 Hz, and the wavelength used were 760 nm and 850 nm. The EEG system (microEEG, BioSignal Group, US) was used with 21 measurement channels (F3, Fz, F4, Fc5, Fc1, Fc2, Fc6, C5, C3, C1, Cz, C2, C4, C6, Cp5, Cp1, Cp2, Cp6, P3, Pz, and P4) referenced to Fcz. The ground electrode was placed frontally on Fpz. The electrodes used were standard Ag/AgCl ones and the EEG signals were sampled at *f*_*eeg*_ = 250 Hz. EEG electrodes and fNIRS probes were mounted on an extended EEG cap (actiCAP 128, Brain Products GmbH, Germany) and fNIRS sources and detectors were placed maximum at 3.4 cm from each other, in order to ensure good quality signals. The presence of hair, that represents the main limitation for fNIRS recordings, was treated with a cautious placement of the optodes after moving the hair aside with optically conductive gel. NIRStar software (NIRx Medizintechnik GmbH, Germany) was used both to acquire the data and to check the quality of the signals before starting the experiments. Presentation software (Neurobehavioral Systems, US) was used to guide the subjects during the experiment, to synchronize the signals, and to keep the log of the different phases of the trials. Fig 1 shows the location of EEG electrodes and fNIRS probes.

### Data Analysis

EEG and fNIRS signal processing was performed offline, but all the methods involved were chosen to be translatable in an online setup. EEG signals were first of all filtered in the *μ* and *β* band, namely 8–12 Hz and 18–25 Hz, respectively, with 4th order IIR Butterworth filters. The band-pass of the filters was chosen in order to identify Sensory Motor Rhythms (SMR) components, which are de-synchronized during motor tasks and re-synchronysed when the motor task is stopped: the modulation of motor-related rhythms is known as Event-Related De-synchronization (ERD) and Event-Related Synchronization (ERS) [23]. In the current study, Slow Cortical Potentials (SCPs) were not evaluated as in [24], because an exact timing of the movements was not available for the experimental setup did not include EMG measurements. fNIRS raw signals, representing the light attenuation in the two wavelengths, were converted into concentrations changes of oxy-hemoglobin (HbO) and deoxy-hemoglobin (HbR) by means of the Modified Beer-Lambert Law [25, 26]. HbO and HbR signals were then filtered with a 4th order IIR Butterworth filter between 0.01 and 0.2 Hz. Differently from other studies, such as [20, 21], fNIRS signals were processed with a high pass filter in order to eliminate slow drifts of the baseline in the signals. After the filtering step, both EEG and fNIRS time series of each measurement channel were normalized by subtracting the mean and dividing by the standard deviation of the entire signals. Eventually, EEG and fNIRS time series were synchronized using Presentation events.

EEG signals were processed using Common Spatial Patterns (CSP) method [8, 9]. CSP perform a subject-dependent and supervised decomposition that enhances the discriminability between two classes. Given *N* channels, the CSP algorithm output is a set of spatial filters *W* whose first components have maximum variance for *C*1 and minimum for *C*2, and the last ones have maximum variance for *C*2 and minimum for *C*1. After the estimation of the optimal spatial filters, the signals are projected on the spatial filters, before extracting features. Let $x\left(t\right)\in R^{N}$ be the pre-processed signals at time *t*, where *N* is the number of measurement channels:

$$\begin{array}{c} x_{CSP}\left(t\right)=W^{T}x\left(t\right) \end{array}$$ (1)

where $x_{CSP}\left(t\right)\in R^{N}$ is the set spatially filtered signals at time *t*. The estimate of the optimal spatial filters is performed by simultaneously diagonalizing the two covariance matrices representing the two classes (*∑*_*C*1_ and *∑*_*C*2_):

$$\begin{array}{c} \{\begin{array}{c} W^{T}\sum_{C1}W=\Lambda_{C1}\\ W^{T}\sum_{C2}W=\Lambda_{C2} \end{array} \end{array}$$ (2)

where *Λ*_*C*1_ and *Λ*_*C*2_ are the diagonal matrices containing the eigenvalues. It is important to notice that *W* can be re-scaled in order to have *Λ*_*C*1_ + *Λ*_*C*2_ = *I*, so that signals belonging to *C*1 have maximum variance when projected on the first components of *W* and minimum when projected on its last components, and signals of class *C*2, on the contrary, have an opposite behavior.

Due to the tendency of CSP of over-fitting small training datasets [27, 28]—25 trials per class can be considered a small dataset for BCI applications—CSP were applied after regularizing the estimated covariance matrices for each class. Regularization means adding *a-priori* knowledge at the covariance matrix estimation step [28] so that it does not adhere excessively to the training dataset, but it generalizes over new testing data. As regularization technique we chose the Generic Learning, which shrinks the covariance matrices of the 2 classes towards the identity matrix (weighed by a factor *γ*, Eq 3a) and towards a generic covariance matrix *Γ*_*C*_ obtained from all the subjects involved in the study (by a factor *β*, Eq 3b):

$$\begin{array}{ccc} {\tilde{\Sigma }}_{C} & = & \left(1-\gamma \right){\hat{\Sigma }}_{C}+\gamma I \end{array}$$ (3a)

$$\begin{array}{ccc} {\hat{\Sigma }}_{C} & = & \left(1-\beta \right)s_{C}\Sigma_{C}+\beta \Gamma_{C} \end{array}$$ (3b)

where *s*_*C*_ is a constant scalar. Regularized CSP method differs from CSP only for the fact that instead of the sample-based covariance matrices *∑*_*C*1_ and *∑*_*C*2_ in Eq 2, the regularized covariance matrices ${\tilde{\Sigma }}_{C1}$ and ${\tilde{\Sigma }}_{C2}$ are used.

The Generic Learning Regularized Common Spatial Patterns (GLRCSP, which will be named simply RCSP in the remaining of the paper) have been proposed by Lu et al. [29] and they showed improvements in terms of accuracy for small datasets. In order to estimate the *γ* and *β* parameters for each spatial filter estimation, a Genetic Algorithm (GA) was used. The GA aimed at optimizing the 4 parameters involved in each CSP regularization (*γ*_1_, *γ*_2_, *β*_1_, and *β*_2_, where 1 and 2 refer to *C*1 and *C*2) using the accuracy obtained with a 10-fold cross-validation of a Linear Discriminant Analysis (LDA) classifier as fitness function. Features were extracted from time segments of 1 second with 50% overlap between each other. From *x*_*CSP*_(*t*) (Eq 1) band powers were computed by rectifying the signals and low-pass filtering them [30]. A feature vector was made of the concatenation of the average band powers of the first 3 and the last 3 CSP components (which carry most of the discriminative information, as explained by [9]) computed from 3 consecutive time segments, in order to take into account the dynamics of the signals [31]. Since CSP can augment the separability of 2 classes only, for the multi-class recognition 3 different sets of spatial filters were estimated from the data: one for Rest-Task, one for Right-Left, and one for Arm-Hand classification. CSP filters were estimated separately for *μ*- and *β*-filtered signals.

For HbO and HbR signals, two methods were investigated to extract features. The first approach computed features with RCSP, but, differently from the EEG, the range within every time segment was chosen as features instead of the variance of the signal (due to the slow dynamic of fNIRS signals). Also for fNIRS features were extracted from the first 3 and the last 3 CSP components, concatenating 3 consecutive time windows. The second approach adopted to extract features is certainly more straightforward: a feature vector contained the average of the band-passed fNIRS signals and a slope indicator, which was simply the difference between the current time segment average and the one computed from the previous time segment, from every channel. Features were extracted separately from HbO and HbR signals.

The classifier used, as anticipated before, is a LDA. Before training the classifiers, the features derived from both EEG and fNIRS were normalized and log transformed, in order to meet the assumptions of normality and equality of variance on which LDA is based [32, 33]. The classification is thought to be performed in two stages: the first one takes care of the asynchronous paradigm, i.e. classifies whether the user is resting or moving (any movement); the second step is performed only when a movement is detected and it classifies the task between Right-Left and Arm-Hand. The choice of clustering two separate classes into a single *macro* class (e.g. Right-Arm and Right-Hand into Right or Left-Hand and Right-Hand into Hand) had the rationale of trying to overcome the strong over-fitting by doubling the number of trials per class. Classifiers for each recognition step were trained on 7 different sets of features: using *μ*, *β*, HbO, and HbR derived features, using EEG features (*μ* + *β*), fNIRS features (HbO + HbR) and the concatenation of EEG and fNIRS derived features (*μ* + *β* + HbO + HbR). No feature selection method is applied at this stage, because the feature set is small and does not impose computational load; in addition we have taken separate measures against over-fitting. Implementing feature selection is being planned for a subsequent study. The choice of the classification flow is discussed in detail in the Discussions and Conclusion section.

The performance of each classifier was evaluated by means of a 10-fold cross validation. At each iteration, randomly, 90% of the trials served as training set, LDA were estimated and the accuracy, i.e. the ratio between correct predictions and the total number of predictions, was computed on the remaining 10% of the trials, the testing set. Accuracies were computed using features from the interval [+2, +6] s after the task visual cue. For the Rest-Task classifier, Rest features were computed from the interval [−4, 0] s before the task cue presentation. In order to evaluate the classification in a more dynamic way, the trend of the accuracy over the trial was obtained as follows: each testing trial of every validation step was synchronized and clipped between −3 s before the beginning of the task (presentation of the visual cue to the subject, at time 0) and 5 s after the end of the task (presentation of the Rest text). The accuracy was computed for every time segment in the interval by building CSP and classifiers using the entire time period ([−3, +11] s). Then, accuracies were averaged among the 10 steps of the cross-validation, yielding an accuracy signal along the trial for each classifier. Another qualitative representation of the dynamic of the response is shown by the evolution of scalp plots representing the trend of every EEG or fNIRS channels along the trial duration, averaged over all the trials and all subjects. Since the CSP method mixes the information of every channel, in order to visualize the features CSP were not applied: for the EEG the band powers of every channel after laplacian filters were used, while for fNIRS the scalp plots displays the evolution of the features extracted without the use of CSP, namely, average and slope indicators.

## Results

The recognition of Rest or Task mental state is the first step for the development of a BCI with an asynchronous paradigm, in which the user is continuously operating the system. Table 1 shows the performance, in terms of average accuracy among all subjects ± its standard deviation, obtained by predicting the label of each time segment using the corresponding features (1 s time window with 50% overlap). A 100% of accuracy means that all the predictions of the testing sets within the cross-validation step are correct, while 50% is the performance of a random classifier. The highest accuracy for the EEG is obtained when *μ* and *β* features are combined (85.2±4.6%); fNIRS performs better when average and slope features are extracted from the signals and it outperforms EEG both when HbO and HbR are used separately, and when the extracted features are concatenated (92.4±5.3%). The highest accuracy is reached when EEG and fNIRS features are used together (HYB) to train the LDA (94.2±3.4%).

**Table 1:** Accuracy [%] for Rest-Task classification. The values are the average accuracy among the 15 subjects ± the standard deviation. *RCSP* stands for Regularized Common Spatial Patterns (applied with the Generic Learning approach), while *AV-SL* indicates the use of average and slope indicators as fNIRS features.

Re-T	*μ*	*β*	HbO	HbR	EEG	fNIRS	HYB
*RCSP*	78.6±5.7	82.8±5	69.4±4.1	65.9±4	**85.2±4.6**	69.8±4.5	86.2±4
*AV-SL*			90.5±6	89±7.1		**92.4±5.3**	**94.2±3.4**

**Fig 2:**
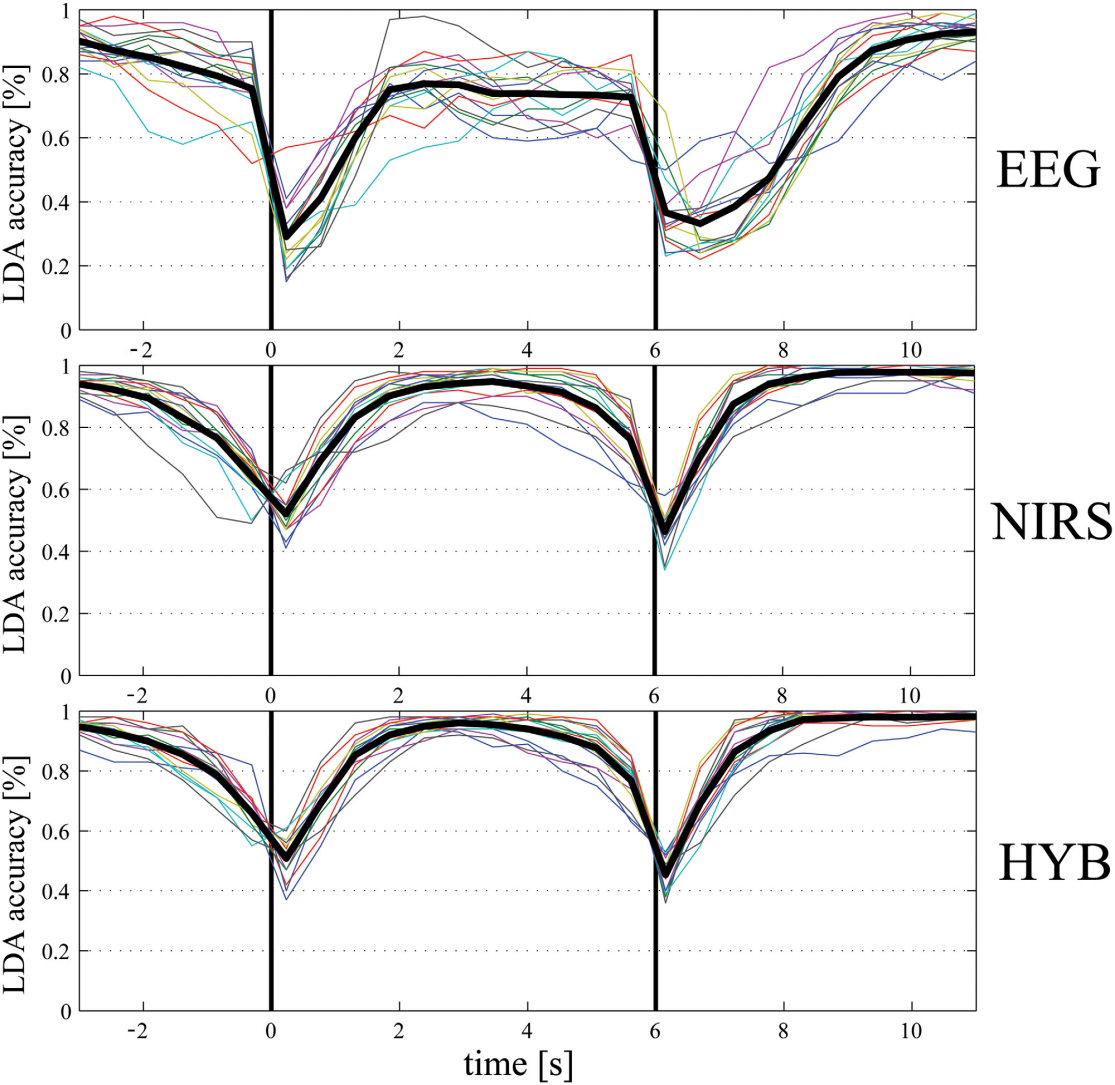
EEG, fNIRS, and HYB Rest-Task classification accuracy [%] for a 1 s moving window with 50% overlap (top: EEG, middle: fNIRS, bottom: HYB). The colored lines represent the different subjects and the black thick line is the average accuracy. The first black vertical line (at time 0 s) is the beginning of the task, while the second one (at time 6 s) is the end of it.

Fig 2, shows the trend of the accuracy along the trial for the EEG (*RCSP*) classifier (top row), the fNIRS (*AV-SL*, i.e. using features representing averages and slopes) one (middle row), and the HYB classifier (EEG + fNIRS, bottom row). The trends reflect the performances shown in Table 2: fNIRS accuracy is higher than EEG one, and the combination of features yields the best performance. The drop of accuracy observed around 0 s and 6 s, i.e. at the beginning and at the end of the task (denoted with the black vertical solid lines in Fig 2), is due to the reaction time for both starting and stopping the movement: the time entire interval [0, +6] s is labeled Task, and the remaining ([−3, 0] s and [+6, +11]) is labeled Rest. However, the accuracy trend reaches its maximum around 2 s after the cues presentation (at time 0 and 6 s) and it is very accurate for the entire duration of the trial, appearing promising for the development of an asynchronous paradigm.

**Table 2:** Accuracy [%] for Right-Left classification.

R-L	*μ*	*β*	HbO	HbR	EEG	fNIRS	HYB
*RCSP*	61±9.8	58.7±7.3	62.2±4.3	60.9±4.6	62.2±8.9	63.1±5.8	67.1±7.4
*AV-SL*			70.6±9.4	65±8.5		70±7.8	**72.2±6.9**

**Fig 3:**
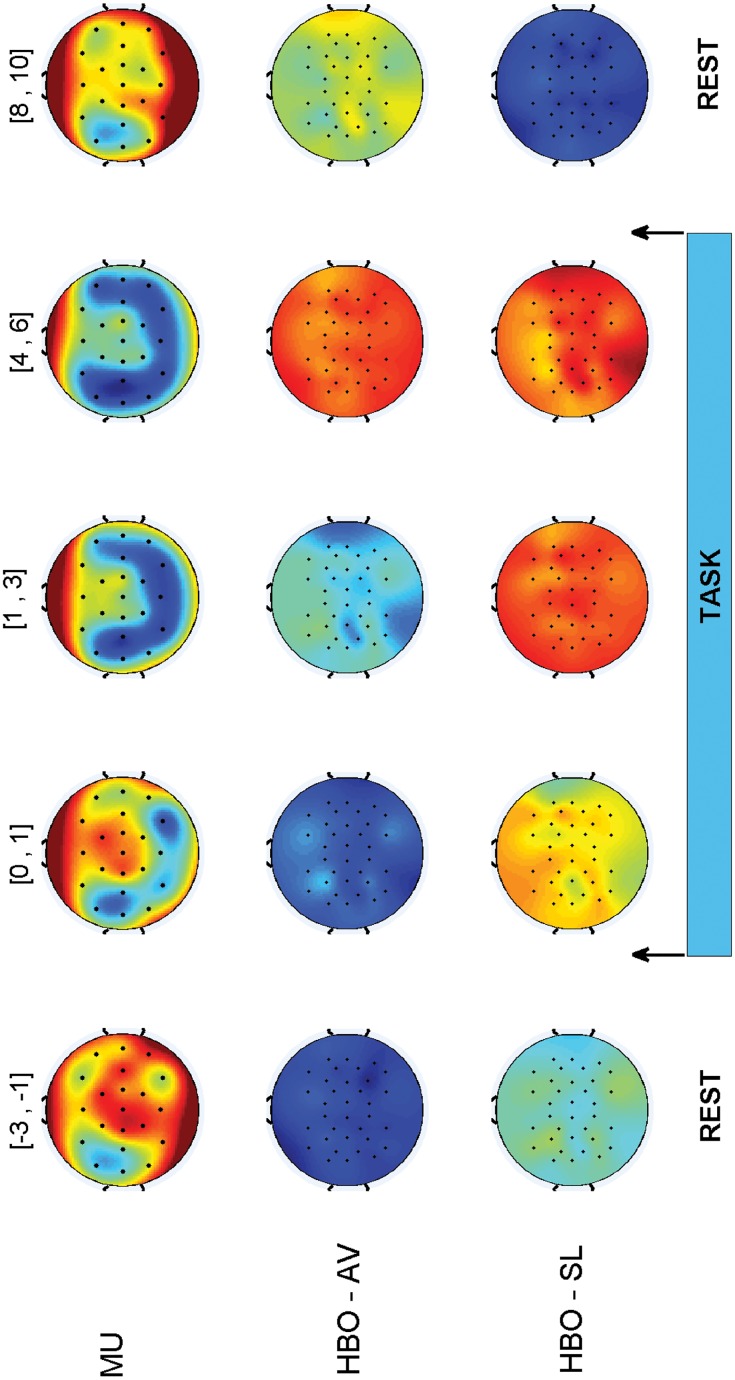
*μ* power, HbO averages, and HbO slopes (top: EEG, middle: HbO-average, bottom: HbO-slope) scalp plots along the trial (values are averaged over all subjects). The values are computed every 1 s with 50% overlap and averaged over the time interval shown on top (e.g. [−3, −1]: values averaged between −3 s and −1 s). The task, as shown by the light blue rectangle at the bottom, starts at 0 s and ends at 6 s.

Fig 3 displays qualitative scalp plots representing the trend of the signals along the trial. The plots are built by averaging the responses of all subjects and all the trials. The first row shows the evolution of EEG band powers for each channel. ERD and ERS can be easily observed (cold colors represent ERD and hot colors stand for ERS): during the task, ERD takes place mainly on motor related channels, while when the task ends the rhythms (in this case *μ*) are re-synchronized (ERS). It is interesting to compare the informative role of fNIRS signals (HbO average and slope are shown in the second and third row, respectively): during the task, the level of oxygenation increases, and then it slowly decreases (HBO—AV). The slope feature appears to play an important role in the early detection of a task: after the planning of the movement ([0, 1] s time interval), the slope significantly increases with respect to the Rest state, and it goes down right after the task. The combination of average and slope feature makes the classification more robust.

### Right-Left and Arm-Hand

After the classification of Task, the movement has to be discriminated between one of the 4 classes. Table 2 contains the performance (average accuracy among subjects ± standard deviation) of the Right-Left classifiers. It is important to notice that no difference between arm or hand movements is considered in this classification. The best performance using features derived from single signal features is achieved using an HbO-based classifier (70.6±9.4%). The EEG performance is lower than the fNIRS one (maximum of 62.2±8.9% when *μ* and *β* derived features are combined) and this is probably because the large amount of over-fitting occurring applying CSP algorithms, due to the very small dataset and despite the regularization technique (this topic is discussed in Discussions and Conclusion section). The combination of EEG and fNIRS provide an improvement in the performance, both by enhancing the average accuracy and by limiting the standard deviation (72.2±6.9%).

**Fig 4:**
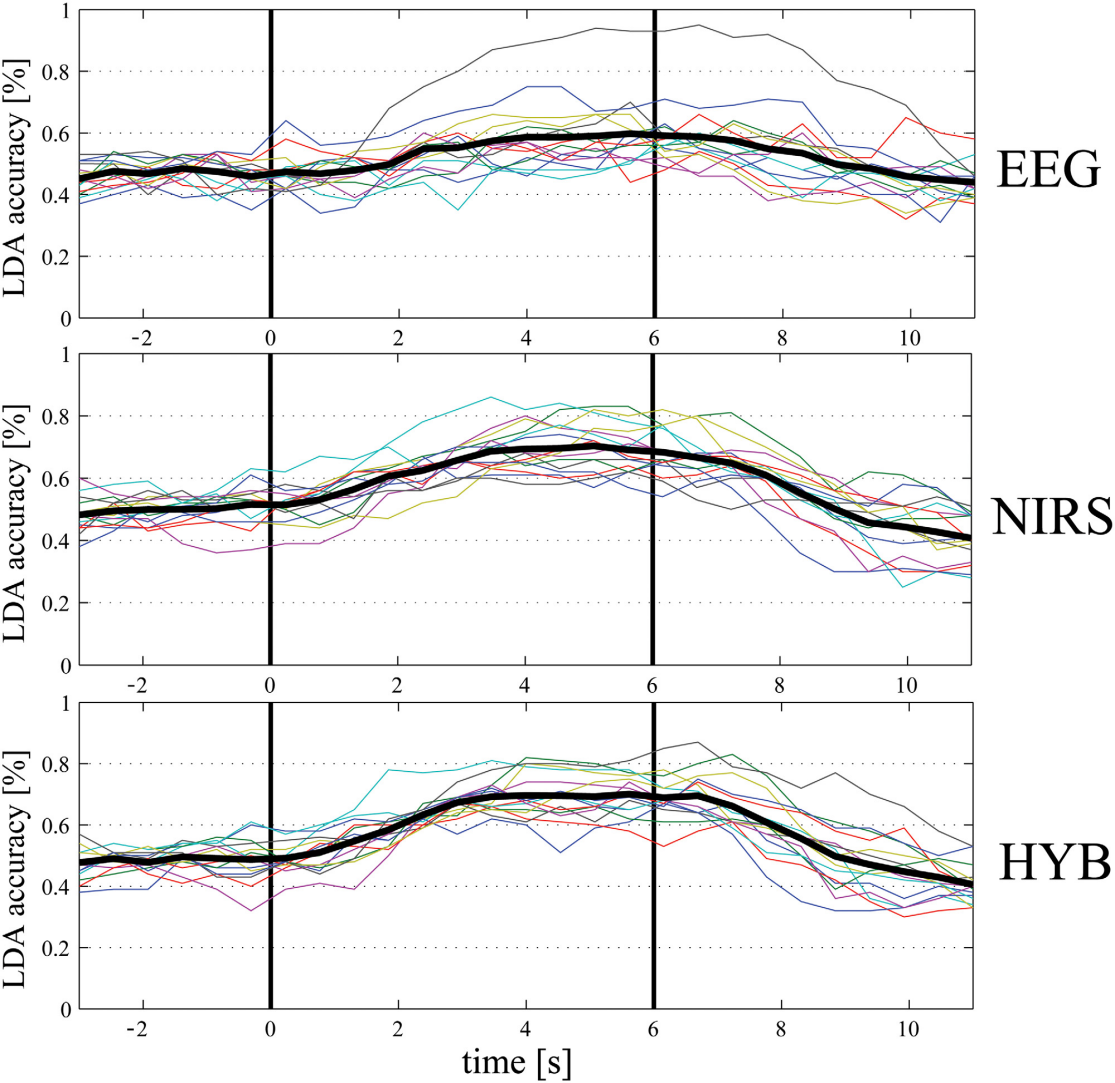
EEG, fNIRS, and HYB Right-Left classification accuracy [%] for a 1 s moving window with 50% overlap (top: EEG, middle: fNIRS, bottom: HYB). The colored lines represent the different subjects and the black thick line is the average accuracy. The first black vertical line (at time 0 s) is the beginning of the task, while the second one (at time 6 s) is the end of it.

The dynamic accuracy of EEG, fNIRS and HYB classifiers is shown in Fig 4. For EEG, RCSP are used, and for fNIRS average and slope features (*AV-SL*). Even for this classification, fNIRS performance is higher than EEG one and the delay in the hemodynamic response observed in [21], is limited in terms of accuracy: the fNIRS-based classifier reaches a steady point of around 70% between 3.5 and 4 s after the stimulus onset (first black vertical line). The use of HbO and HbR slopes along with averages over 1 s time windows, therefore, increases the responsiveness of fNIRS classifiers also for Right-Left recognition.

The final step of classification aims at recognizing Arm or Hand movements. Note that this step does not have to be performed *after* the Right-Left one: the 2 classifiers following the Rest-Task one can be run in parallel and will output one of the 4 classes, when combined. In case of Arm-Hand classifiers, differently than Rest-Task and Right-Left ones, RCSP yields the best performances for fNIRS. As shown in Table 3, *RCSP* approach reaches a higher accuracy than *AV-SL* one for HbO, HbR, and fNIRS derived features. Moreover, fNIRS classifies better than EEG. The highest accuracy is obtained when EEG and fNIRS features using RCSP are combined to build the LDA classifier (83.6±9.6%).

**Table 3:** Accuracy [%] for Arm-Hand classification.

A-H	*μ*	*β*	HbO	HbR	EEG	fNIRS	HYB
*RCSP*	69.3±12.3	65.8±8.1	79.4±8.7	76.7±10.7	71±11.9	80.4±9.1	**83.6±9.6**
*AV-SL*			75.5±8.1	73.4±7.4		76.9±6.4	79.9±7.1

**Fig 5:**
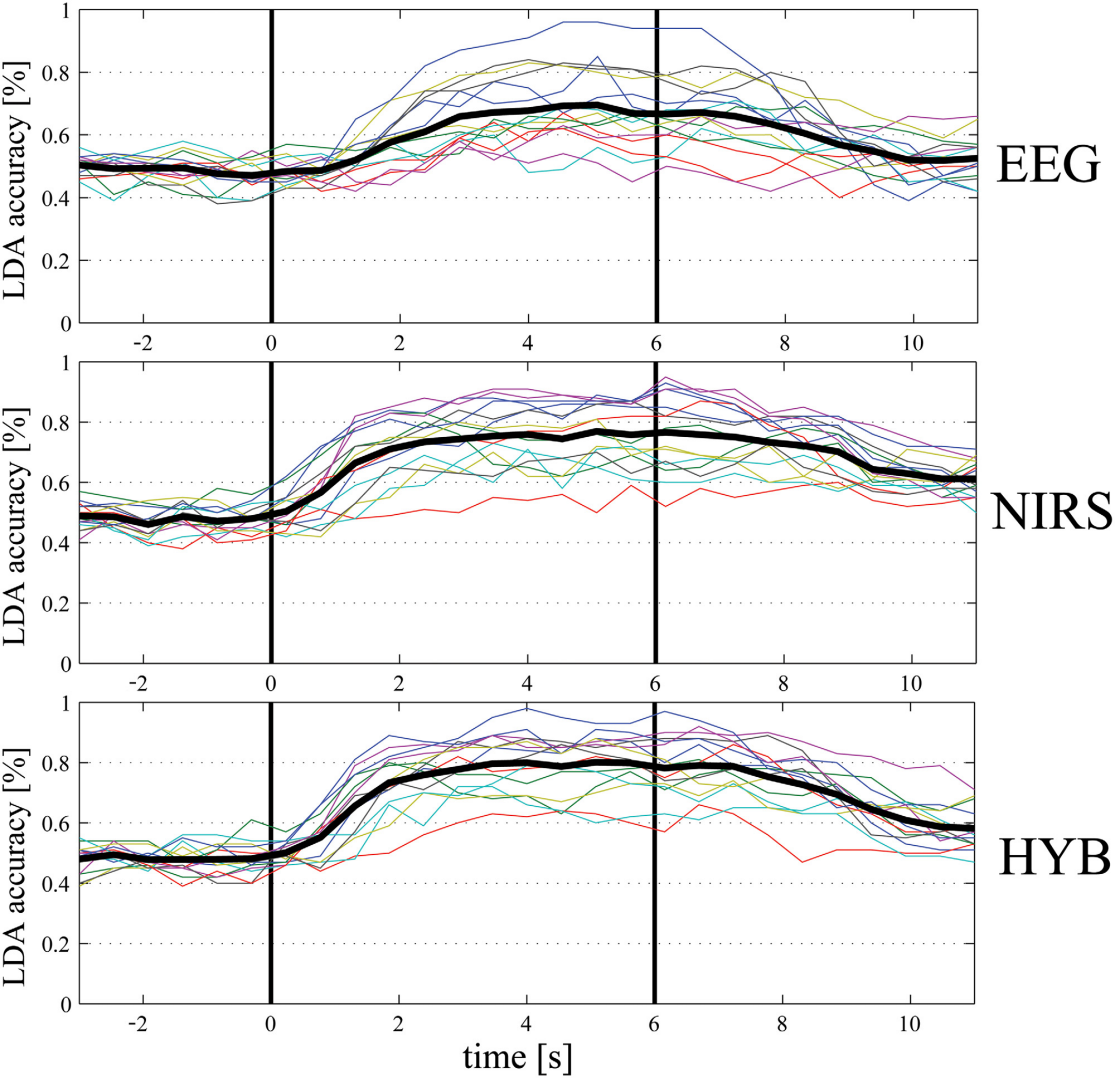
EEG, fNIRS, and HYB Arm-Hand classification accuracy [%] for a 1 s moving window with 50% overlap (top: EEG, middle: fNIRS, bottom: HYB). The colored lines represent the different subjects and the black thick line is the average accuracy. The first black vertical line (at time 0 s) is the beginning of the task, while the second one (at time 6 s) is the end of it.

Regarding the evolution of the classifiers’ performances, from Fig 5 it can be observed that the readiness of fNIRS-based classifiers, on average, is faster using CSP method (second row): a steady value of accuracy around 80% is reached after 2–2.5 s from the task visual cue. The response of the classifier is actually even better when accounting for the reaction time of the subjects. The use of CSP method on fNIRS appears promising for the detection of topographically different cortical activities. As shown by [34], in fact, cortical activity appears in the controlateral region before the movement and becomes bilaterally symmetrical during the actual execution. This effect could explain why the performances in the recognition of Arm-Hand are higher than Right-Left classifiers, as discussed in detail in the Discussions and Conclusion section.

One final comment on Figs 4 and 5: due to the asynchronous paradigm, which triggers the classification of one of the four classes only when Task is detected, the dynamic accuracies plotted are significant if the Rest-Task classifier predicts a task, i.e., as shown in Fig 2, in the interval between 1.5 s and 5.5 s. Elsewhere, in fact, the classification of Right-Left and Arm-Hand would not be performed.

## Discussions and Conclusion

In this paper we reported the performance of an EEG-fNIRS-based BCI in discriminating between a set of motor tasks. In all cases, the accuracy of the hybrid system was higher than the accuracy of a subsystem based on an individual modality (EEG or fNIRS). A recent study has demonstrated that combining fNIRS and EEG enhances the performance of a SMR-based BCI system only in terms of accuracy, due to the slow dynamics of HbO and HbR signals [21]. In the current work we aimed at improving the hybrid BCI design mainly regarding the fNIRS processing, since EEG techniques have been widely developed and well-established. Moreover, we wanted to investigate the capability of such a system in an asynchronous paradigm, in which the user is in continuous communication with the system and to extend the number of classes from 2 (Right-Hand—Left-Hand) to 4 (or 5 if considering Rest as a class). The results showed that fNIRS enhances significantly the performance of EEG alone when detecting a generic Task, yielding an average accuracy of 94.2±3.4% and proving the suitability of the hybrid approach for this purpose. For further classification (Right-Left, Arm-Hand) the use of fNIRS in the adopted experimental setup and procedure (i.e. location and number of channels and number of trials per class) outperformed EEG classifiers, probably due to the EEG relative low spatial configuration (21 recording electrodes, 37 in [21]). The main goal has been the identification of a new set of fNIRS features capable of an early recognition of the different movements. While the average values of HbO and HbR over a time window as features yields an accuracy peak occurring around 6.5–7.5 s after the movement onset (as shown in [21]), the inclusion of slope indicators allowed to anticipate it of around 3 s for Right-Left recognition (peak occurring around 3.5–4 s after movement onset, see Fig 4—second row). The use of RCSP, despite the small dataset and the over-fitting phenomenon described in the next paragraph, resulted in an early response of the Arm-Hand classifiers, in which the accuracy peak was reached, on average, around 2–2.5 s after the task cue (as shown in Fig 5—second row). Clearly, due to the different information carried by EEG and fNIRS, their combination in a hybrid approach is beneficial in terms of robustness of the BCI. It is true, however, that in order to fully develop and evaluate the multi-class capability of the system, the binary classifiers should be combined to output only one of the 4 classes at every time segment. The combination of Right-Left and Arm-Hand classifiers could also account for the confidence of the binary prediction. The main drawback of the hybrid system, however, is in the time required for setting up both the system. An interesting option to tackle this issue could be opting for EEG dry electrodes, which have been already applied in the BCI research [35]. Currently, though, fNIRS technology is not as easy-to-use as EEG one, but portable systems are already available (e.g. NIRSport, NIRx Medizintechnik GmbH, Germany).

While controlled hand movements likely produce manageable movement artifacts, full arm movements may have a higher contribution in terms of artifacts. However, the band-pass filters used both for EEG and fNIRS should attenuate motion artifacts. Moreover, since the movements were self-paced and features were computed over a 1 s time segment with 50% overlap, the artifacts will tend to cancel out over a time average. For EEG only, a threshold of ±30 *μ*V was empirically identified and used as saturation in order to limit the effects of possible parasitic EMG activity. The results showed a better performance in Arm-Hand classification than Right-Left one. It is possible that part of the discrimination power is due to motion artifact occurring during full arm movement itself. However, Right-Left overt movements, as shown in [34], strongly activate both hemispheres and this can be the reason why in this case Right-Left performance is around 10% lower than Arm-Hand one. Future studies will involve EMG recordings in order to evaluate the presence of artifacts and the possible correlation with the system performance for motor execution.

The experimental design and procedure strongly affected the performance, mainly for EEG. It is well known that EEG suffers from low spatial resolution, due to the volume conduction of the tissues between the recording site, on the scalp, and the cortical electrical activity. Volume conduction is the main issue for EEG source localization [11, 12]. The use of high resolution EEG can no doubt partially overcome this problem, but in this study a light setup has been preferred also in order to mimic and resemble a possible clinical application. The second factor that conditioned the performance of the system has been the number of trials per class available, with respect to the use of CSP. CSPs are deeply affected by over-fitting, i.e. they excessively adhere to the dataset used to estimate them and have poor generalization over new observations. Being a *supervised* approach that makes use of labeled data, the limited amount of training data plays a very important role. This is probably the main reason for the EEG lower performance with respect to other studies involving a higher number of trials, e.g. [4] used 60 trials per class, [21] had 48 executed movements and 100 imagined trials per class, and 140 trials per class in [29]. The choice of grouping classes in Right-Left and Arm-Hand is also motivated by the small dataset: by grouping two classes together, in fact, a higher number of trials per class was obtained (50 trials for Right, Left, Arm, and Hand classes). On the other hand the clustering in *macro* classes can diminish the generalization of each class in physiological terms.

In this study we presented results only about executed movements, which clearly represents a limitation for BCI applications. Nevertheless, simultaneous recording of electrical and hemodynamic activities have proven a strict correlation between overt movement and motor imagery in terms of topology [34, 36]; therefore, the study of motor execution before motor imagery can give important information on the processes underlying motor tasks. As part of the experiments we also collected data on motor imagery tasks with a basic EEG-based feedback which gives the subject a visual information about the detection of a generic task. Preliminary results showed that the performance of motor imagery was acceptable in Rest-Task classification (85.8±7.2% of accuracy), but not even comparable to the motor execution one in terms of Right-Left and Arm-Hand classification (63.4±7.5% and 60.8±4.4%, respectively). It should be emphasized that all subjects involved in the experiments had no previous experience in motor imagery. In order to be able to reach good performance in motor imagery, [22] states that subjects need to train for 1 to 4 hours with a visual feedback informing the user whether his/her imagery strategy is correctly classified. Future studies will involve the development of a hybrid feedback, involving both EEG and fNIRS measurements, built on classifiers trained on the data collected for the current work. Although it has been shown that HbO and HbR amplitude changes during motor imagery tasks are smaller than in motor executed tasks [37], we think that with a proper feedback and training subjects could achieve acceptable performance in terms of accuracy, as in [21], and that the use of the proposed features for fNIRS would result in a faster response in motor imagery too.

Finally, we believe that the methods and features in this study could invigorate the use of fNIRS, or fNIRS in combination with EEG, in BCI research. Our future work will include the validation of the results obtained and the investigation of the performance of the entire system using a bigger dataset (more trials per class) and possibly increasing the EEG resolution. Moreover, a real-time evaluation of the performance in terms of Information Transfer Rate (ITR, measured in bits/min), would objectively asses the capability of the asynchronous BCI to communicate with an application and provide an alternative output of the central nervous system.
